# Serum VEGF and CGRP Biomarkers: Relationships with Pain Intensity, Electric Pain, Pressure Pain Threshold, and Clinical Symptoms in Fibromyalgia—An Observational Study

**DOI:** 10.3390/ijms242115533

**Published:** 2023-10-24

**Authors:** Rosa Mª Tapia-Haro, Francisco Molina, Alma Rus, Antonio Casas-Barragán, María Correa-Rodríguez, Mª Encarnación Aguilar-Ferrándiz

**Affiliations:** 1 Department of Physical Therapy, Faculty of Health Sciences, University of Granada (UGR), 18016 Granada, Spain; rtapia@ugr.es (R.M.T.-H.); fjmolina@ugr.es (F.M.); antoniocb@ugr.es (A.C.-B.); e_aguilar@ugr.es (M.E.A.-F.); 2 Instituto de Investigación Biosanitaria ibs.GRANADA, 18012 Granada, Spain; mrus@ugr.es; 3 Department of Cell Biology, Faculty of Sciences, University of Granada (UGR), 18016 Granada, Spain; 4 Department of Nursing, Faculty of Health Sciences, University of Granada (UGR), Ave. de la Ilustración, 60, 18016 Granada, Spain

**Keywords:** fibromyalgia, pain, clinical manifestations, biomarkers, vascular endothelial growth factor, calcitonin gene-related peptide

## Abstract

Fibromyalgia (FM) is a multifactorial syndrome, mainly characterized by chronic widespread pain, whose physiopathology is yet to be determined. Reliable biomarkers for FM and how they are associated with the symptomatology have not yet been identified. We aimed to examine the relationships among serum vascular endothelial growth factor (VEGF) and calcitonin gene-related peptide (CGRP) levels with clinical manifestations and pain-related variables in women with FM. We conducted an observational case study with forty-seven women diagnosed with FM. Serum VEGF and CGRP levels were spectrophotometrically analyzed. We used questionnaires to measure the impact of FM and the degree of central sensitization, fatigue, and anxiety. We also assessed pain intensity, electric pain threshold and magnitude, and pressure pain threshold (PPT) in tender points. The linear regression analysis adjusting for age, menopause status, and body mass index showed that serum VEGF levels were significantly associated with the PPTs of non-dominant trapezius (β = 153.418; *p* = 0.033), non-dominant second metacarpal (β = 174.676; *p* = 0.008) and dominant tibialis anterior (β = 115.080; *p* = 0.049) in women with FM. We found no association between serum CGRP levels and the variables measured (*p* ≥ 0.152). Our results suggest that VEGF may be related to pain processing in patients with FM.

## 1. Introduction

Fibromyalgia (FM) is a complex syndrome mainly characterized by chronic widespread musculoskeletal pain defined as pain persisting for more than 3 months without an underlying pathology or obvious organic damage [1,2,3]. The presence of chronic pain in these patients usually coexists with a low pain tolerance, hyperalgesia and allodynia [4]. Additionally, other additional symptoms such as fatigue joint stiffness, paresthesia, swelling in the hands, headaches, sleep disturbances, anxiety, cognitive dysfunction, and depression may be present [1,2,3,4,5].

FM is one of the main reasons for patient referral to rheumatology services and the third most frequent musculoskeletal condition, after lumbar pain and osteoarthritis [1,5,6]. FM affects the quality of life of patients and is associated with significant socioeconomic costs [7]. The average worldwide prevalence of FM is 2.7%, ranging from 0.4 to 9.3%. The average prevalence in the American continent is 3.1%, in Europe it is 2.5%, and it is 1.7% in Asia [8]. In the Spanish population, the prevalence is 2.4% [4]. FM is more common in women (4.2%) than in men (0.2%), with a female/male ratio that can range from 21:1 [4] to 1:3 [8].

The physiopathology of FM is not yet clearly defined due to its complexity and multifactorial nature, which makes its diagnosis and treatment difficult [3,4,9,10]. In this regard, biochemical, metabolic, immune system, and genetic factors have been described as possible mediators that could influence the development of fibromyalgia [11]. Different peripheral and central mechanisms have been proposed to be involved in the physiopathology of FM, including the theory of the sensitization of the central nervous system, dysfunction of the autonomic nervous system (ANS), impairment in the microcirculation, and small fiber neuropathy [2,9,12,13,14]. Several lines of research suggest central sensitization (CS) as the main pathophysiological mechanism of FM [7,15,16,17]. The possible origin of FM is established as a dysfunction in the processing of nociceptive stimuli at the level of the central nervous system (CNS) [17]. The symptoms of patients with FM are due to functional and morphological changes in the brain structures that process pain in the CNS [7,15,16,17]. CS could explain these changes, associated with glial activation due to neuroinflammation triggers [18]. Peripheral nociceptors send pain impulses to neurons in the dorsal horn of the spinal cord in conditions of chronic pain. Sustained activation of nociceptive fibers stimulates the release of neurotransmitters (substance P, glutamate, calcitonin gene-related peptide, and aspartate). The increase in neurotransmitters triggers postsynaptic responses by hyperexciting N-methyl-D-aspartate (NMDA) receptors [16,17]. Therefore, through ascending pathways, postsynaptic transmission to supraspinal structures (thalamus, hypothalamus, anterior cingulate cortex, insular cortex, limbic system and somatosensory cortex) is increased [15,16,17,18]. For all these reasons, through CS, sustained pain can produce a state of hyperexcitability in the CNS [16,17,18]. In this line, studies with functional magnetic resonance imaging in the resting state have described functional changes related to CS in patients with FM, showing greater neuronal activation at rest in pain processing regions, so chronic pain could induce changes in brain processes, even in the absence of an external stimulus [18]. Also, the physiological mechanism of pain inhibition (descending pathways of the corticoreticular system, hypothalamus and brainstem) is altered in CS [16,17,18]. The release of neurotransmitters that inhibit brain function, such as serotonin, norepinephrine, enkephalins, and g-aminobutyric acid (GABA) is modified [16]. Therefore, these changes in ascending pathways and altered descending modulation may lead to CS in patients with FM [16,17]. Evidence also suggests that ANS dysfunction may be key in the generation and maintenance of chronic pain, as well as symptoms in FM. People with FM have increased sympathetic activity and decreased parasympathetic activity compared to healthy subjects [9,12]. In this regard, a systematic review conducted in 2013 [19] to determine differences in heart rate variability between patients with FM, chronic fatigue syndrome, and healthy subjects, reported that people with FM showed a lower heart rate compared to healthy subjects, an increase in sympathetic activity, and a blunted autonomic response to stressors [19].

Other studies found microcirculatory disturbances to be a possible cause of FM symptoms. Consequently, evidence mentions that patients with FM have fewer capillaries and an abnormally reduced diameter, as well as density, of digital capillaries compared to healthy people. Subjects with FM also have greater tortuosity of their capillaries to compensate for the decreased microcirculation [13]. Moreover, the study by Albrecht et al. [14] reported modifications in peripheral vascular responses in people with FM compared to healthy controls. FM subjects showed a significant increase in sensory innervation and sympathetic in the arteriovenous anastomoses (AVAs) in the hypothenar eminence of their hands, with a higher proportion of vasodilator sensory fibers in comparison with the vasoconstrictor sympathetic fibers. This increased innervation in AVAs caused an increase in the production of pain-related vasodilator peptides. Thus, the excessive AVAs innervation could be a possible cause of pain, tenderness, fatigue, and CS in people with FM [14]. Furthermore, it has been described that patients with FM may present mitochondrial dysfunction and oxidative stress [10]. Mitochondrial abnormalities could contribute to symptoms such as fatigue and pain. There appears to be a relationship between oxidative stress, pain, and the pathogenesis of FM [10]. In line with this, previous studies also reported higher levels of proinflammatory cytokines IL-6, IL-8, IL-1, and tumor necrosis factor in FM patients [10,11].

In recent years, there has been a growing interest in searching for potential biomarkers that aid in the diagnosis and clarify the physiopathology of this syndrome [2,5,11,18]. A current systematic review [18] highlights the existence of various studies that have attempted to determine possible biomarkers that may be related to pain processing in FM patients. In this sense, possible alterations in metabolites such as glutamate, substance P, nerve growth factor, brain-derived neurotrophic factor, Mu opioid receptor, cytokines, and neuropeptides, among others, have been analyzed. However, none of the metabolites analyzed have been shown to have results reliable enough to be validated as a possible diagnostic biomarker of FM [18]. In line with this, recent evidence indicates that vascular endothelial growth factor (VEGF), a molecule that plays a key role in regulating microvascular circulation, may have a nociceptive role in the sensory nervous system. In fact, alterations in VEGF expression have been found to be present in pathologies associated with the development of chronic pain [20]. Recent studies have also shown that CGRP plays a fundamental role in the regulation of vascularization and pain processing [21,22,23]. CGRP may reportedly be involved in the development of central and peripheral sensitization, acting as an excitatory neurotransmitter that facilitates nociceptive transmission, contributing to the development and maintenance of pain in chronic conditions [22,23].

Since both VEGF and CGRP are vasoactive molecules involved in chronic pain modulation and processing, we hypothesize that they may be related to pain and other clinical symptoms in patients with FM. The aim of this study was to examine the relationships among serum VEGF and CGRP levels with pain-related variables and the main FM clinical manifestations in women diagnosed with this syndrome.

## 2. Results

### 2.1. Study Variables in Women with FM

**Figure 1:**
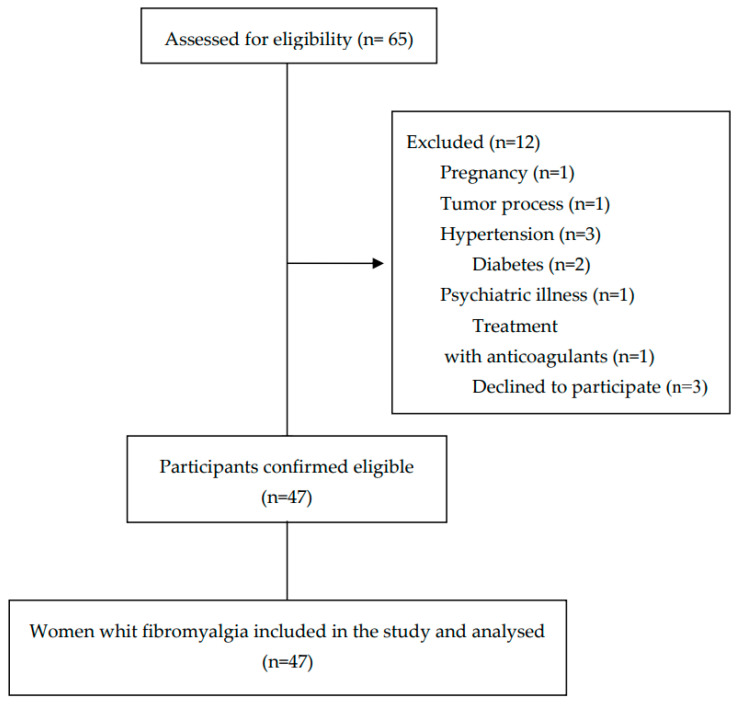
Flow diagram of the participants throughout the study.

Sixty-five participants were initially recruited to participate in the study, and forty-seven women diagnosed with FM were finally selected based on the established selection criteria. A flow diagram of the selection of participants throughout the study is shown in Figure 1.

The sociodemographic data, clinical characteristics, pain measurements, and serum levels of VEGF, as well as the CGRP of the participants, are presented in Table 1.

### 2.2. Associations of Serum VEGF Levels with FIQ-R, CSI, MFI, BAI, VAS, Electric Pain Threshold, Electric Pain Magnitude, and PPTs in Women with FM

The associations between serum VEGF levels and the clinical and pain-related variables of the participants are shown in Table 2. After adjustment for age, body mass index, and menopause status, significant associations were found among serum VEGF levels and non-dominant trapezius PPT (β = 153.418, 95% CI [12.877, 293.958], *p* = 0.033), non-dominant second metacarpal PPT (β = 174.676, 95% CI [47.910, 301.442], *p* = 0.008), and dominant tibialis anterior PPT (β = 115.080, 95% CI [0.510, 229.650], *p* = 0.049). In addition, the following associations approached statistical significance: serum VEGF levels and CSI (β = −7.586, 95% CI [−15.607, 0.435], *p* = 0.063), electric pain magnitude (β = 131.936, 95% CI [−21.741, 285.613], *p* = 0.090), non-dominant occiput PPT (β = 105.729, 95% CI [−13.284, 224.741], *p* = 0.080), non-dominant supraspinatus PPT (β = 85.572, 95% CI [−7.209, 177.771], *p* = 0.070), non-dominant epicondyle (β = 124.098, 95% CI [−22.241, 270.437], *p* = 0.094) and non-dominant knee PPT (β = 68.053, 95% CI [−6.586, 142.691], *p* = 0.073).

### 2.3. Associations of Serum CGRP Levels with FIQ-R, CSI, MFI, BAI, VAS, Electric Pain Threshold, Electric Pain Magnitude, and PPTs in Women with FM

The associations between serum CGRP levels and the clinical features, as well as pain-related variables, of the patients with FM are presented in Table 3. We did not find any statistically significant association between serum CGRP levels and clinical variables (*p* ≥ 0.152) nor pain variables (*p* ≥ 0.186) in women with FM after adjusting for age, menopausal status, and body mass index.

## 3. Discussion

Although the physiopathology of FM remains unknown, several factors have been proposed to be possible causes for the generation and maintenance of chronic pain and other FM symptoms, including impaired pain processing at the central and peripheral level and alterations of the ANS, small fibers, or microcirculation. VEGF and CGRP are molecules involved in the regulation of both microvascular circulation and pain. However, the role that they play in FM remains to be determined. In this study, we focused in examine the relationships among serum VEGF and CGRP levels with pain variables and clinical manifestations in patients with FM.

Our results revealed significant positive associations among serum VEGF levels and some PPTs (non-dominant trapezius, non-dominant second metacarpal, and dominant tibialis anterior) in women with FM. Additionally, associations of VEGF levels with CSI, electric pain magnitude, non-dominant occiput PPT, non-dominant supraspinatus PPT, non-dominant epicondyle, and non-dominant knee PPT approached statistical significance. Our results indicate that VEGF may be associated with the PPTs of several tender points in women with FM, suggesting that this molecule may be related to pain processing in these patients. To our knowledge, there are only three studies available that compared the levels of VEGF between healthy subjects and patients with FM, showing contradictory results [24,25,26]. The study of Blanco et al. [24] reported lower serum levels of VEGF in patients with FM than in healthy subjects, while the other two studies [25,26] did not find significant differences in serum VEGF levels between patients with FM and controls. Moreover, studies examining correlations between VEGF and clinical characteristics in patients with FM have shown conflicting results. In line with our results, Karadağ et al. [26] found a significant positive association between serum VEGF levels and the number of tender points in patients with FM. On the contrary, the study of Kim et al. [25], which compared serum VEGF levels between FM patients with and without diverse clinical features, found significant differences in overall stiffness, showing that patients with FM who had lower serum VEGF levels had higher levels of stiffness. Previous studies [27,28] have reported that pain in patients with FM could be caused by altered microvascular circulation with a decreased blood flow at tender points, which can lead to local hypoxia and reduced aerobic capacity [26,27,28]. When tissue hypoxia occurs, VEGF, which acts by binding two specific tyrosine kinase receptors, VEGF receptor-1 and VEGF receptor-2, is upregulated to regulate microvascular circulation, control blood vessel growth, prevent endothelial damage, and induce angiogenesis [20,26]. In addition, VEGF may produce hyperexcitability in sensory neurons, causing sensitization to pain [29]. Thus, and agreeing with our results, altered levels of VEGF could be related to microvascular alterations and muscle damage in patients with FM, leading to symptoms such as pain, muscle weakness, and stiffness [24,26].

Our results also showed that serum CGRP levels were not significantly associated with the pain and clinical features measured in women with FM. CGRP is a 37-amino-acid peptide that is present in the central and peripheral nervous systems as well as non-neural tissues such as epithelial cells, lymphocytes, and adipocytes. CGRP is found mainly in sensory fibers C and A, located in the brain, intestine, and perivascular nerves. It regulates the functioning of the cardiovascular, digestive, and sensory systems, and is involved in the pathogenesis of various pain-related syndromes [21,22,30,31]. CGRP is a potent vasodilator with pro-inflammatory effects, which is implicated in the development of neurogenic inflammation and inflammatory, as well as neuropathic, pain [21,22]. Nociceptive activation of C fibers and release of neuropeptides (substance P, CGRP, and prostanoids) results in neurogenic inflammation. Thus, the development and maintenance of pain in FM could be caused by neuroinflammation at the peripheral tissue level [18]. To date, only one study has evaluated CGRP levels in patients with FM, finding higher CGRP levels in patients with FM than in healthy subjects [23]. These authors recognized the lack of an objective measurement of pain level and the lack of a correlation between pain level and CGRP level as limitations of the study [23]. To our knowledge, this is the first study that assesses the associations of CGRP with pain-related variables and clinical manifestations in women diagnosed with FM. A recent systematic review reported the association between CGRP levels and different conditions of somatic, visceral, neuropathic, and inflammatory pain, suggesting that CGRP may be involved in nociceptive pathways in various pain conditions [32]. Although our results show that CGRP does not seem to be associated with FM symptoms, further studies would be needed to clarify the role of CGRP in the physiopathology and symptomatology of FM.

The lack of reliable biomarkers in FM makes the early and adequate diagnosis of FM difficult [18]. The diagnosis of FM remains exclusively clinical today. The main symptom for the diagnosis of FM is the pain that patients present. Pain perception is a complex variable to measure, since it is very subjective and could be influenced by multiple factors, which makes it more difficult to make an accurate diagnosis [33]. Furthermore, uncertainty about the etiology, symptoms, diagnosis, treatment, and outcome, due to the absence of biomarkers, increases frustration and dissatisfaction in patients suffering from FM [18]. Uncertainty about the illness accompanied by intermittent pain predicted greater difficulty coping with the illness and negatively affected treatment [34]. Uncertainty was also related to higher levels of anxiety [35] and greater comorbidity, as well as psychiatric problems [36,37]. Hence, given the complexity of this syndrome, being able to establish FM biomarkers would be key to making an accurate diagnosis and even more specific, as well as concrete, treatments.

The present study has some limitations that should be recognized. Firstly, although the sample size in our study was adequately powered, future studies with a larger sample size are needed to corroborate our findings and extrapolate the data. Secondly, the design of the study is observational, which means that our results must be carefully interpreted. Finally, even though we have used validated instruments to evaluate variables related to pain, pain perception is a complex variable to measure, since pain perception is very subjective and could be influenced by multiple factors; therefore, our results should be interpreted with caution.

Our study also shows strengths, since it is the first research that examines two vasoactive biomarker molecules involved in nociception, CGRP, and VEGF, trying to determine their possible associations with pain variables and clinical manifestations in patients with FM. This study opens up new research possibilities in relation to the role that these biomarkers may have in the physiopathology and symptomatology of FM. Therefore, further research in this area will be necessary to identify specific biomarkers that assist in the diagnosis and treatment of FM.

## 4. Materials and Methods

### 4.1. Study Design and Participants

We performed an observational cases study with forty-seven women diagnosed with FM. The Ethical Committee of Research of Granada (Spain) approved the study (No. 1718-N-18), which was conducted in accordance with the amended version of the Declaration of Helsinki, 2013.

The patients were recruited were from two Andalusian Associations of Fibromyalgia (Association of Fibromyalgia of Granada (AGRAFIM) and the Association of Fibromyalgia of Jaén (AFIXA),Granada and Jaén, Spain) between September 2019 and December 2019. The volunteers were screened according to the demographic and clinical data obtained in a first inter-view carried out in the Faculty of Health Sciences of the Universities of Granada and Jaén. The inclusion criteria were as follows: (1) diagnosis of FM from a rheumatologist of the Public Health System of Andalusia (Spain) according to the ACR criteria [38]; (2) age range 18–70 years; and (3) female gender. Based on the data on the prevalence of FM by gender, it is higher in women, and for this reason, only men were included in the study [39,40]. The exclusion criteria were as follows: (1) pregnancy or breastfeeding; (2) the presence of chronic diseases such as cancer, hypertension, or diabetes mellitus; (3) the presence of renal, cardiac, or hepatic insufficiency; (4) neurological disorders; (5) psychiatric illness; and (6) treatment with vasoactive drugs, corticosteroids, agonist-antagonist opioid receptors, anticoagulants, or estrogens. We obtained the written informed consent from all of the participants.

### 4.2. Outcome Measures

In the first visit, the volunteers were asked to fill out a questionnaire to obtain sociodemographic and clinical data such as age, weight, height, menopause status, medical history, and medication consumed. In the second visit, blood was drawn from the selected participants, and they then completed several questionnaires to measure the impact of FM, and the degree of CS, fatigue, and anxiety. Finally, we measured the pain intensity, electric pain threshold and magnitude, and pressure pain threshold (PPT) in tender points.

#### 4.2.1. Questionnaires

To assess the degree of the severity and effects of FM, we used the Spanish version of the Revised Fibromyalgia Impact Questionnaire (FIQ-R) [41]. This questionnaire consists of 21 items. The score ranges from 0 to 100, with higher scores indicating greater severity. The Spanish version of the FIQ-R has a good internal consistency (Cronbach’s alpha = 0.91) [41]. We employed the Spanish version of the Central Sensitization Inventory (CSI) to obtain information about CS-related symptoms [42]. This is a self-reported inventory with 25 items, and the frequency of each symptom is recorded by a five-point Likert scale. The score ranges from 0 to 100, where higher scores indicate major severity and frequency of symptoms [43,44]. The cut-off score to determine the presence of CS is 40. The inventory shows high internal consistency, with a Cronbach’s alpha of 0.88 [44]. We evaluated the psychological aspects and common symptoms of anxiety with the Spanish version of the Beck Anxiety Inventory (BAI) [45]. The questionnaire consists of 21 items that assess the severity of anxiety from 0 points (no anxiety) to 3 points (a lot of anxiety). The score ranges from 0 to 63, where higher scores indicate higher levels of anxiety. The Spanish version of the BAI has shown a high internal consistency (Cronbach’s alpha = 0.93) [45,46]. We evaluated the fatigue with the Spanish version of the Multidimensional Fatigue Inventory (MFI) [47]. This questionnaire is divided into five subscales with four questions in each one, and has a score range from 20 to 100, with higher scores indicating higher degree of fatigue. The Spanish version of the questionnaire has shown a good internal consistency (Cronbach’s alpha = 0.93) [47,48].

#### 4.2.2. Pain Assessment

We used a Visual Analogue Scale (VAS) to assess the global intensity of pain, consisting of a 10 cm line where 0 means no pain and 10 means the worst pain imaginable [49]. This instrument has shown high sensibility and specificity in the FM population, with a good internal consistency (Cronbach’s alpha = 0.71 to 0.91) [49,50].

We also measured pain intensity with an electric stimulation device, a Pain Matcher (PM) (Cefar-Compex Scandinavia Inc, Medical AB, Lund, Sweden). This generates an increasing electric current [51]. To create the electrical stimulus, participants held the carbon rubber electrodes of the device with a firm grip between their thumb and the index finger [51,52]. Two pain measures were obtained; the pain threshold and the pain magnitude. For pain threshold value, participants were to release the grip as soon as they had a sensation of pain. For pain magnitude value, participants released the grip when the pain sensation in their fingers matched the intensity of their global musculoskeletal pain [51,52]. Three measurements were made, and the mean was calculated. The Pain Matcher has shown good test–retest reliability (95% confidence interval = 0.39–0.14) [51]. We used an FDIXTN digital algometer (Wagner Instruments, Greenwich, CT, USA) to assess the PPTs. We applied pressure at a rate of 1 kg/s. Each participant informed the examiner when the sensation of pressure changed to pain [53]. We measured PPTs bilaterally over the 11 locomotor points that the ACR determined for FM (occiput, C5-C6 zygapophyseal joint, trapezius, supraspinatus, second rib, epicondyle, second meta-carpal, greater trochanter, gluteus, knee, and anterior tibialis). We performed three measurements in each point, with a 30 second resting period, and calculated the mean. This method has shown good internal consistency, with a Cronbach’s alpha of 0.94–0.98 [54] and an intraclass correlation coefficient of 0.91 [54].

#### 4.2.3. Blood Collection and Measurement of Serum VEGF and CGRP Levels

The same specialized professional collected all blood samples, at the same time of day, to avoid circadian variations in FEV and CGRP levels. We obtained the blood samples from the antecubital vein into an anticoagulant-free tube (BD Vacutainer LH PST II Advance, Ref. 367374; Becton Dickinson, Franklin Lakes, NJ, USA). Blood was allowed to clot for 30 min at room temperature and the tube was then centrifuged at 3500 revolutions per minute (rpm) (Avanti J-30I; Beckman Coulter, Brea, CA, USA) for 5 min at 4 °C to obtain serum. Levels of CGRP and VEGF were determined in serum via an enzyme linked immunosorbent assay (ELISA) following the manufacturer’s recommendations. Specifically, we used a CGRP (human) ELISA kit, reference: #A05481.96; wells; Bertin Bioreagent; and a Human Vascular Endothelial Cell Growth Factor A ELISA Kit, catalogue No.: E-EL-H0111, Elabscience.

### 4.3. Statistical Analysis

The sample size was calculated using Ene 3.0 software (GlaxoSmithKline, Rockbille, MA, USA). To obtain a power (1-β error) of 0.80, with an alpha significance level (α error) of 0.05, and based on the results of CGRP-like activity levels in chronic pain patients [55], the estimated minimum sample size necessary to include in the study is 19 participants.

We used SPSS Statistics Version 24 for Windows (IBM Corporation, Armonk, NY, USA) for data analysis. The normality of the variables was verified with the Kolmogorov–Smirnov test (α value = 0.05). Sociodemographic and clinical variables were tested by a one-sample *t*-test with a 95% confidence interval (CI) (α value = 0.05). The data for continuous variables were expressed as the mean ± standard deviation (SD) and for categorical variables as frequency (%). We performed a multiple linear regression analysis to check associations among serum VEGF levels and CGRP levels with FM clinical features (FIQ-R, CSI, MFI, BAI, EVA, electric pain threshold and magnitude, and PPTs). Since age, body mass index, and menopause status are known confounders that can influence symptoms in FM patients [6,56,57,58], we adjusted the analysis by taking into account these factors. The results of the linear regression analysis were expressed as beta estimate (β) with 95% CI and *p*-value. *p* < 0.05 was considered statistically significant.

## 5. Conclusions

We have found significant associations between serum VEGF levels and the PPTs of several tender points in women with FM, suggesting that this molecule may be related to pain symptoms in these patients. However, no significant associations were found between serum CGRP levels and FM symptoms, suggesting that this molecule may not be involved in pain variables in FM patients. Therefore, future studies along this line would be necessary to determine specific biomarkers that help in the diagnosis and treatment of FM.

## Figures and Tables

**Table 1:** Sociodemographic data, clinical characteristics, pain measurements, and biological parameters in women with fibromyalgia.

Outcomes		Women Diagnosed with Fibromyalgia (n = 47)	
		Mean ± SD/Frequency (%)	95% CI
Age (years)		56.06 ± 6.41	[54.18, 57.95]
Height (cm)		157.83 ± 5.37	[156.25, 159.41]
Weight (kg)		68.21 ± 10.09	[65.24, 71.17]
BMI (kg/cm^2^)		27.49 ± 4.59	[26.14, 28.84]
Menopause status			
Postmenopausal		38 (80.9)	
Premenopausal		9 (19.1)	
FIQ-R		73.24 ± 13.20	[69.32, 77.16]
CSI		68.87 ± 11.55	[65.48, 72.26]
MFI		79.68 ± 9.79	[76.81, 82.56]
BAI		32.60 ± 8.45	[30.12, 35.08]
VAS (mm)		74.47 ± 16.66	[69.58, 79.36]
Electric pain threshold (mA)		5.75 ± 3.09	[4.83, 6.67]
Electric pain magnitude (mA)		11.33 ± 7.89	[8.99, 13.68]
Pressure pain thresholds (kPa)			
Occiput	D	1.03 ± 0.42	[0.82, 1.24]
	ND	0.96 ± 0.71	[0.75, 1.17]
Zygapophyseal joint	D	1.21 ± 0.82	[0.97, 1.45]
	ND	1.15 ± 0.84	[0.90, 1.39]
Trapezius	D	1.07 ± 0.81	[0.83 1.31]
	ND	0.99 ± 0.63	[0.80, 1.17]
Supraspinatus	D	1.42 ± 1.13	[1.09, 1.76]
	ND	1.39 ± 0.88	[1.12, 1.64]
Second rib	D	0.96 ± 0.53	[0.80, 1.11]
	ND	0.88 ± 0.48	[0.74, 1.02]
Epicondyle	D	1.07 ± 0.67	[0.88, 1.23]
	ND	1.05 ± 0.60	[0.87, 1.22]
Second metacarpal	D	1.28 ± 0.79	[1.05, 1.52]
	ND	1.15 ± 0.64	[0.96, 1.34]
Greater trochanter	D	2.17 ± 1.06	[1.85, 2.48]
	ND	2.22 ± 1.13	[1.98, 2.55]
Gluteus	D	2.09 ± 1.67	[1.59, 2.59]
	ND	1.97 ± 1.33	[1.58, 2.36]
Knee	D	1.81 ± 1.17	[1.46, 2.15]
	ND	1.99 ± 1.11	[1.66, 2.32]
Anterior tibialis	D	1.99 ± 1.33	[1.60, 2.38]
	ND	1.97 ± 1.18	[1.62, 2.32]
VEGF (pg/mL)		354.29 ± 269.69	[275.11, 433.48]
CGRP (pg/mL)		36.96 ± 5.83	[35.25, 38.67]

Note: Data are expressed as mean ± SD (standard deviation) for quantitative variables and as frequency (%) for qualitative variables. Abbreviations: CI (confidence interval); BMI (body mass index); FIQ-R (revised fibromyalgia impact questionnaire); CSI (central sensitization inventory); MIF (multidimensional fatigue inventory); BAI (Beck anxiety inventory); VAS (visual analogue scale); mA (milliamperes); kPa (kilopascals); D (dominant); ND (non-dominant); VEGF (vascular endothelial growth factor); and CGRP (calcitonin gene-related peptide).

**Table 2:** Beta estimates, confidence intervals, and *p*-values for the association between VEGF and clinical features in women with fibromyalgia.

Variable		Women Diagnosed with Fibromyalgia (n = 47)		
		Serum VEGF Levels		
		β	95% CI	*p*-Value
FIQ-R		−2.929	[−9.511, 3.653]	0.374
CSI		−7.586	[−15.607, 0.435]	0.063
MFI		−6.163	[−14.242, 1.920]	0.131
BAI		−6.323	[−15.810, 3.164]	0.186
VAS (mm)		−0.232	[−19.276, 18.813]	0.981
Electric pain threshold (mA)		130.464	[−56.148, 317.075]	0.166
Electric pain magnitude (mA)		131.936	[−21.741, 285.613]	0.090
Pressure pain thresholds (kPa)				
Occiput	D	81.418	[−45.012, 207.849]	0.201
	ND	105.729	[−13.284, 224.741]	0.080
Zygapophyseal joint	D	41.557	[−59.295, 142.408]	0.410
	ND	77.398	[−17.147, 171.942]	0.106
Trapezius	D	76.786	[−29.710, 183.283]	0.153
	ND	153.418	[12.877, 293.958]	0.033 *
Supraspinatus	D	67.588	[−47.595, 182.771]	0.243
	ND	85.281	[−7.209, 177.771]	0.070
Second rib	D	82.572	[−75.866, 241.011]	0.299
	ND	44.153	[−136.470, 224.776]	0.624
Epicondyle	D	94.297	[−35.924, 224.517]	0.151
	ND	124.098	[−22.241, 270.437]	0.094
Second metacarpal	D	46.024	[−55.289, 147.336]	0.364
	ND	174.676	[47.910, 301.442]	0.008 *
Greater trochanter	D	29.501	[−55.326, 114.329]	0.486
	ND	62.310	[−18.607, 143.228]	0.128
Gluteus	D	92.195	[−32.859, 217.248]	0.144
	ND	52.960	[−11.039, 116.960]	0.102
Knee	D	37.270	[−36.219, 110.758]	0.312
	ND	68.053	[−6.586, 142.691]	0.073
Anterior tibialis	D	115.080	[0.510, 229.650]	0.049 *
	ND	57.224	[−12.185, 126.634]	0.104

* Significance level: *p* < 0.05. Note: Beta (β) represents the regression coefficient. Adjusted for age, body mass index, and menopause status. Abbreviations: VEGF (vascular endothelial growth factor); CI (confidence interval); FIQ-R (revised fibromyalgia impact questionnaire); CSI (Central Sensitization Inventory); MIF (Multidimensional fatigue Inventory); BAI (Beck anxiety inventory); VAS (visual analogue scale); mA (milliamperes); kPa (kilopascals); D (dominant); and ND (non-dominant).

**Table 3:** Beta estimates, confidence intervals, and *p*-values for the association between CGRP and clinical features in women with fibromyalgia.

Variable		Women Diagnosed with Fibromyalgia (n = 47)		
		Serum CGRP Levels		
		β	95% CI	*p*-Value
FIQ-R		−0.101	[−0.241, 0.039]	0.152
CSI		−0.014	[−0.173, 0.146]	0.864
MFI		−0.085	[−0.263, 0.092]	0.339
BAI		−0.035	[−0.244,0.174]	0.737
VAS (mm)		0.064	[−0.46, 0.175]	0.248
Electric pain threshold (mA)		−2.680	[−6.701, 1.342]	0.186
Electric pain magnitude (mA)		0.473	[−2.947, 3.893]	0.781
Pressure pain thresholds (kPa)				
Occiput	D	0.607	[−2.173, 3.386]	0.622
	ND	0.190	[−2.477, 2.857]	0.887
Zygapophyseal joint	D	−0.222	[−2.418, 1.973]	0.839
	ND	−0.847	[−2.938, 1.245]	0.419
Trapezius	D	−0.501	[−2.854, 1.852]	0.669
	ND	−1.166	[−4.135, 1.803]	0.433
Supraspinatus	D	−0.684	[−3.204, 1.837]	0.587
	ND	−0.014	[−2.222, 1.935]	0.890
Second rib	D	−1.536	[−4.970, 1.899]	0.372
	ND	−1.759	[−5.634, 2.116]	0.365
Epicondyle	D	−0.327	[−3.210, 2.555]	0.820
	ND	−1.216	[−4.274, 1.843]	0.427
Second metacarpal	D	1.222	[−1.021, 3.466]	0.277
	ND	0.638	[−2.336, 3.611]	0.667
Greater trochanter	D	0.813	[−0.978, 2.603]	0.364
	ND	0.153	[−1.613, 31.919]	0.862
Gluteus	D	−0.255	[−3.026, 2.516]	0.854
	ND	0.159	[−1.268, 1.586]	0.823
Knee	D	0.179	[−1.428, 1.785]	0.823
	ND	0.062	[−1.614, 1.739]	0.940
Anterior tibialis	D	−0.384	[−2.975, 2.207]	0.766
	ND	−0.212	[−1.759, 1.335]	0.783

Significance level: *p* < 0.05. Note: Beta (β) represents the regression coefficient. Adjusted for age, body mass index, and menopause status. Abbreviations: CI (confidence interval); FIQ-R (revised fibromyalgia impact questionnaire); CSI (Central Sensitization Inventory); MIF (Multidimensional fatigue Inventory); BAI (Beck anxiety inventory); VAS (visual analogue scale); mA (milliamperes); kPa (kilopascals); D (dominant); and ND (non-dominant).

## Data Availability

Data are available on reasonable request. The datasets used and/or analyzed during the current study are available from the corresponding author on reasonable request.
